# A Genome Scan for Positive Selection in Thoroughbred Horses

**DOI:** 10.1371/journal.pone.0005767

**Published:** 2009-06-02

**Authors:** Jingjing Gu, Nick Orr, Stephen D. Park, Lisa M. Katz, Galina Sulimova, David E. MacHugh, Emmeline W. Hill

**Affiliations:** 1 Animal Genomics Laboratory, School of Agriculture, Food Science and Veterinary Medicine, College of Life Sciences, University College Dublin, Belfield, Dublin, Ireland; 2 The Breakthrough Breast Cancer Research Centre, Chester Beatty Laboratories, The Institute of Cancer Research, London, United Kingdom; 3 University Veterinary Hospital, School of Agriculture, Food Science and Veterinary Medicine, College of Life Sciences, University College Dublin, Belfield, Dublin, Ireland; 4 Vavilov Institute of General Genetics, Russian Academy of Sciences, Moscow, Russia; 5 Conway Institute of Biomolecular and Biomedical Research, University College Dublin, Dublin, Ireland; Pasteur Institute, France

## Abstract

Thoroughbred horses have been selected for exceptional racing performance resulting in system-wide structural and functional adaptations contributing to elite athletic phenotypes. Because selection has been recent and intense in a closed population that stems from a small number of founder animals Thoroughbreds represent a unique population within which to identify genomic contributions to exercise-related traits. Employing a population genetics-based hitchhiking mapping approach we performed a genome scan using 394 autosomal and X chromosome microsatellite loci and identified positively selected loci in the extreme tail-ends of the empirical distributions for (1) deviations from expected heterozygosity (Ewens-Watterson test) in Thoroughbred (n = 112) and (2) global differentiation among four geographically diverse horse populations (F_ST_). We found positively selected genomic regions in Thoroughbred enriched for phosphoinositide-mediated signalling (3.2-fold enrichment; P<0.01), insulin receptor signalling (5.0-fold enrichment; P<0.01) and lipid transport (2.2-fold enrichment; P<0.05) genes. We found a significant overrepresentation of sarcoglycan complex (11.1-fold enrichment; P<0.05) and focal adhesion pathway (1.9-fold enrichment; P<0.01) genes highlighting the role for muscle strength and integrity in the Thoroughbred athletic phenotype. We report for the first time candidate athletic-performance genes within regions targeted by selection in Thoroughbred horses that are principally responsible for fatty acid oxidation, increased insulin sensitivity and muscle strength: ACSS1 (acyl-CoA synthetase short-chain family member 1), ACTA1 (actin, alpha 1, skeletal muscle), ACTN2 (actinin, alpha 2), ADHFE1 (alcohol dehydrogenase, iron containing, 1), MTFR1 (mitochondrial fission regulator 1), PDK4 (pyruvate dehydrogenase kinase, isozyme 4) and TNC (tenascin C). Understanding the genetic basis for exercise adaptation will be crucial for the identification of genes within the complex molecular networks underlying obesity and its consequential pathologies, such as type 2 diabetes. Therefore, we propose Thoroughbred as a novel in vivo large animal model for understanding molecular protection against metabolic disease.

## Introduction

As flight animals and grazers the wild ancestors of modern horses were naturally selected for speed and the ability to traverse long distances. Since horses were domesticated on the Eurasian steppe some 6,000 years ago [1] they have been selected for strength, speed and endurance-exercise traits. This process has been uniquely augmented in Thoroughbred horses, which for four centuries have been subject to intense artificial selection for system-wide structural and functional adaptations that contribute to athletic performance phenotypes. As a result Thoroughbreds possess a range of extreme physiological characteristics enabling both high anaerobic and aerobic metabolic capabilities. In comparison to other athletic species of similar size, the aerobic capacity or maximal oxygen uptake (VO_2max_) of Thoroughbreds is superior (>200 ml O_2_/kg/min) [2], [3], and is achieved by a remarkable oxygen carrying capacity and delivery facilitated by structural and functional adaptations involving the respiratory and cardiovascular systems [5]. Specifically, some of these adaptations include a large lung volume, high maximum haemoglobin concentration and cardiac output as well as a large muscle mass (approximately 55%) to body weight ratio, high skeletal muscle mitochondrial density and oxidative enzyme activity and large intramuscular stores of energy substrates (primarily glycogen) in which equivalent concentrations are only achieved in human skeletal muscle after carbohydrate loading [6], [7], [8], [9]. Similar to humans, the VO_2max_ in horses is usually limited by oxygen supply to the mitochondria rather than by mitochondrial oxidative capacity [10], with the respiratory system in horses unable to meet the metabolic demands of exercising muscle [11], [12].

Although the physical and physiological adaptations contributing to elite athleticism in Thoroughbred are well described, the genes contributing to an athletic phenotype have not yet been identified. Domestic animal species provide valuable opportunities to identify genes underlying phenotypes that have been strongly selected because discrete breeds have arisen relatively recently from a small number of founder animals. The Thoroughbred population is a closed population established in the 16^th^ and 17^th^ centuries from crosses between local Galloway and Irish hobby horses with imported Eastern stock [13]. As with many domesticates, the Thoroughbred originates from a small number of founders; just one founder stallion contributes to 95% of paternal lineages and ten founder mares account for 72% of maternal lineages [14]. However, despite a limited number of founders and strong selection for racetrack performance some 35% of variation in performance is heritable [15], [16]. These population demographics coupled with intense recent selection for athleticism offer a unique opportunity to identify genomic contributions to exercise-related traits.

A number of approaches may be taken to identify genes underlying phenotypic adaptations. Whereas a candidate gene approach requires *a priori* knowledge of gene function and linkage mapping requires information about familial relationships as well as access to samples from large numbers of relatives, hitchhiking mapping using population genetics-based approaches evaluates the effects of natural or artificial selection across whole genomes in populations of unrelated individuals that have been subjected to differential selection pressures for the trait or traits of interest [17], [18], [19], [20]. Although it is generally considered that microsatellites themselves will not be subject to selection, loci closely linked to the microsatellites will influence their population genetic behaviour [17], [18], [19], [21], [22], [23]. Therefore we have employed a hitchhiking mapping approach to identify signatures of positive selection in the Thoroughbred genome and to localise genomic regions containing genes influencing exercise-related phenotypes.

## Results

### Identification of selected genomic regions

**Figure 1:**
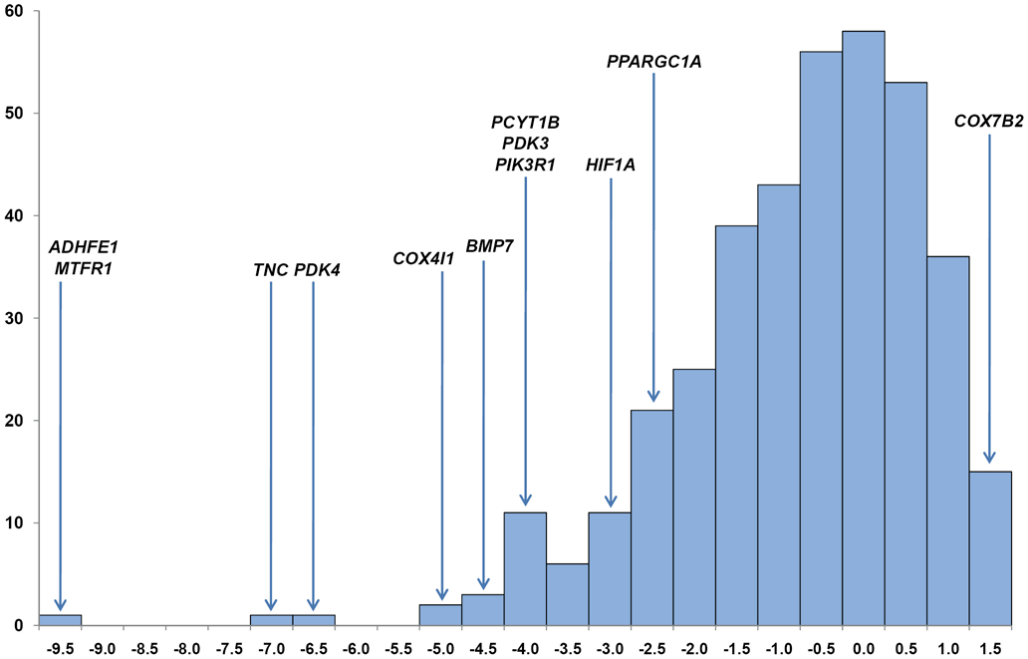
Empirical distribution for Dh/sd. The empirical distribution for Dh/sd scores (horizontal axis) from the Ewens-Watterson test. The vertical axis indicates the number of loci with the corresponding Dh/sd score. The positions in the distribution of the regions that have been subject to selection in Thoroughbred are indicated by the gene symbols for the strongest candidate genes. Genes in the negative tail-end of the distribution may have been subject to positive selection. Genes in the positive end of the distribution may have been subject to balancing selection.

The Ewens-Watterson test statistic identifies positively selected marker loci by evaluating significant deviation from expected heterozygosity (Dh/sd) at individual loci in a single population [24], [25]. Loci experiencing the greatest selection pressures can be found in the extreme tail-ends of empirical distributions [26]. Considering the empirical distribution for Dh/sd (Figure 1) we identified 18 loci in the tail-ends of the distribution (*P*<0.01) in Thoroughbred representing <5% of all loci. At the negative end of the distribution 17 loci had Dh/sd<−3.5 (*P*<0.01) and 55 had Dh/sd<−2.0 (*P*<0.05). Whereas loci lying at the negative end of the Dh/sd distribution have been subject to positive selection, the positive end of the distribution indicates the effects of balancing selection where two or more alleles are maintained at higher frequency than expected under neutral genetic drift. One of fifteen loci with Dh/sd>1.5 deviated significantly (*P*<0.01) from expectation. Three (*COR101*, *NVHEQ040* and *AHT023*) of the selected loci also had significant deviations from neutrality in Connemara and one locus (*AHT023*) was monomorphic in Akhal-Teke.

**Figure 2:**
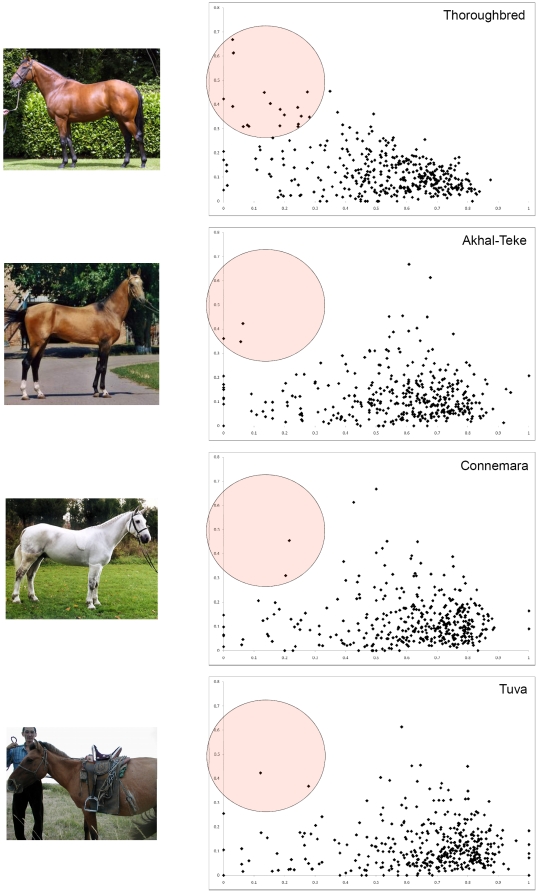
*F*_ST_ versus heterozygosity. *F*_ST_ (vertical axis) versus heterozygosity (horizontal axis) plots for Thoroughbred, Akhal-Teke, Connemara and Tuva at 394 genome-wide microsatellite loci.

**Figure 3:**
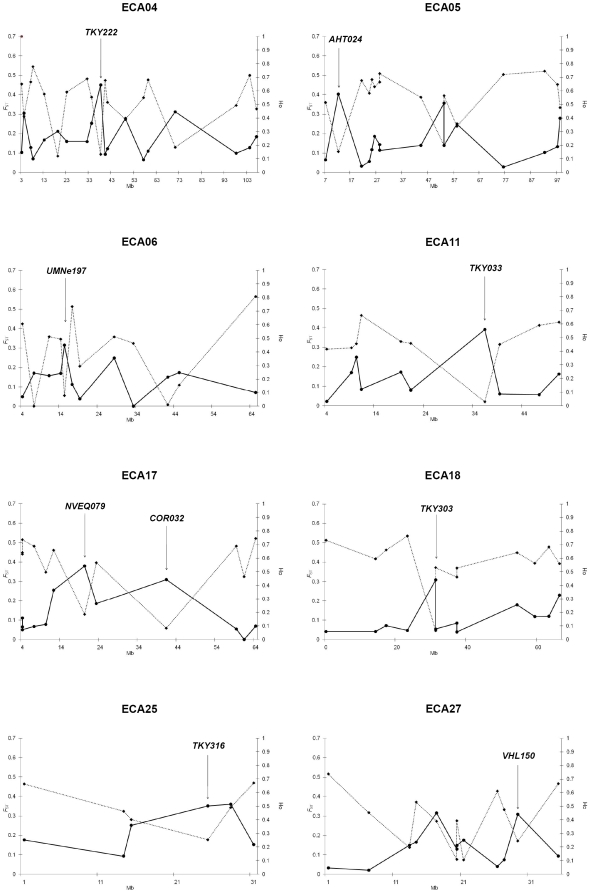
Chromosome-wide *F*_ST_ versus Thoroughbred heterozygosity. *F*_ST_ (solid line) versus Thoroughbred heterozygosity (dashed line) plots across chromosomes for the nine highest *F*_ST_ regions with significant (*P*<0.05) deviations from expected heterozygosity (Dh/sd) in Thoroughbred. Loci defining selected genomic regions are highlighted. Left vertical axis: *F*_ST_; Right vertical axis: Thoroughbred heterozygosity; Horizontal axis: chromosome position (Mb).

**Figure 4:**
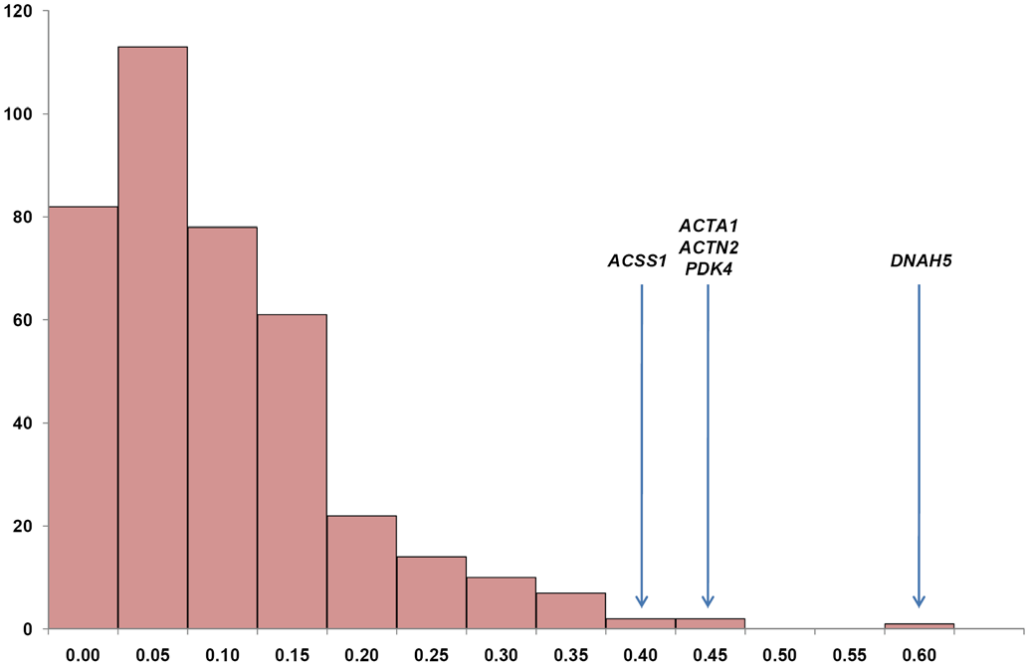
Empirical distribution for *F*_ST_. The empirical distribution for global differentiation, *F*_ST_ (horizontal axis). The vertical axis indicates the number of loci with the corresponding *F*_ST_. The positions of the regions that have been subject to positive selection in Thoroughbred are located in the tail-end of the distribution and are indicated by the gene symbols for the strongest candidate genes.

Studies in other species have shown positively selected genes to have unusually large differences among populations and therefore targets of positive selection may also be identified at the extreme of the distribution for global *F*_ST_ [17], [26], [27], [28], [29]. The Ewens-Watterson test is particularly useful for highly polymorphic loci, such as microsatellites, because it is a function of the number of observed alleles. The *F*_ST_ statistic however does not rely on loci to be highly polymorphic and by evaluating differences among populations rather than in single populations complements the use of the Ewens-Watterson test. We therefore used a combined *F*_ST_ versus *Ho* approach to identify genomic regions that have been targets for positive selection in Thoroughbred. Plotting *F*_ST_ versus *Ho* at each locus in each population revealed a cluster of outlier loci with high *F*_ST_ and low *Ho* unique to Thoroughbred (Figure 2). The mean global *F*_ST_ was 0.12, much lower than has been reported for dog breeds where the mean *F*_ST_ calculated from single nucleotide polymorphism (SNP) data is 0.33 [30]. Over all loci mean *Ho* was lowest in Thoroughbred (*Ho* = 0.536); for the other populations surveyed: *Ho* = 0.587 (Akhal-Teke), *Ho* = 0.630 (Connemara) and *Ho* = 0.672 (Tuva) (Table 1). Another indicator of population genetic diversity, the mean number of alleles sampled per locus, was not lowest in Thoroughbred (4.6 SEM±0.09) but in Akhal-Teke (4.0 SEM±0.08) and was greatest in Tuva (5.0 SEM±0.10). When all populations were considered together the mean number of alleles per locus was 6.7 SEM±0.13. Population differentiation may arise because of random genetic drift and *F*_ST_ may be influenced by unequal sample sizes. Therefore, using a less stringent significance threshold (*P*<0.05) for the Ewens-Watterson test we identified nine of the high *F*_ST_ loci that also had a deficiency in heterozygosity in Thoroughbred. Inter-population *F*_ST_ and Thoroughbred *Ho* was plotted across chromosomes containing the nine most strongly selected loci (Figure 3). Four loci met the more stringent significance (*P*<0.01) in the Ewens-Watterson test. Three loci were common to both tests and one was just below the threshold (Dh/sd = −3.5) for inclusion by the Ewens-Watterson test (Dh/sd = −2.9). The empirical distribution for *F*_ST_ is shown in Figure 4. Of the highest ranked loci in the Ewens-Watterson test, two were almost significant (*COR008* and *AHT006*, *P*<0.1), two were significant (*NVHEQ079* and *TKY316*, *P*<0.05) and one was highly significant (*TKY222*, *P*<0.01) for the *F*_ST_ test.

**Table 1:** Population genetic diversity.

Population	Geographic origin	*n*	*Ho*	Ave. no. alleles sampled/locus	SEM
Thoroughbred	United Kingdom and Ireland	112	0.536	4.6	0.092
Akhal-Teke	Turkmenistan	18	0.587	4.0	0.077
Connemara	Ireland	17	0.630	4.5	0.086
Tuva	Republic of Tuva	17	0.672	5.0	0.099

Horse population origins, number of individuals genotyped (*n*), average observed heterozygosity (*Ho*), mean number of alleles sampled per locus and standard error of mean (SEM).

### Functional ontologies in selected regions

Normally, selection does not act on microsatellites *per se*, but rather on genes to which they are linked by proximity. Therefore, the closest flanking loci meeting neutral expectations were used to define the regions harbouring putative selected genes; although we expect selected genes to lie closer to the central locus than either flanking loci. Linkage disequilibrium is expected to decay within 1–2 Mb in Thoroughbred [31], however a strong selective effect could sweep loci that are located considerably further away. We interrogated the horse genome sequence (EquCab2.0) for genes in the highest ranked regions deviating from expected heterozygosity in Thoroughbred. This analysis included the outlier *F*_ST_ regions confirmed as selected regions in the Ewens-Watterson test as these were the best candidates to contain genes under selection in Thoroughbred. In a list of 765 genes located in the 19 regions we searched for gene ontology (GO) terms that may be important for exercise-related athletic phenotypes. GO Biological Processes, Cellular Compartments and Molecular Functions were returned for 592 genes using the *Homo sapiens* GO database [32]. Genes with functions in 67 GO Biological Processes and 32 GO Molecular Functions (Table S2) were found significantly more frequently than expected (*P*<0.05) for all searched (functionally annotated) genes (*n* = 15,360). GO terms that may have functional relevance for selection in Thoroughbred are listed in Table 2.

**Table 2:** Overrepresented gene ontologies (Dh/sd).

GO Biological Process	GO Identifier	Number of genes	*P*	Fold enrichment
Phosphoinositide-mediated signalling	GO:0048015	13	0.000	3.2
Insulin receptor signalling pathway	GO:0008286	7	0.002	5.0
G-protein signalling, coupled to IP3 second messenger (phospholipase C activating)	GO:0007200	10	0.003	3.4
Spermatogenesis	GO:0007283	18	0.005	2.1
Skeletal development	GO:0001501	18	0.007	2.0
Porphyrin biosynthetic process	GO:0006779	4	0.035	5.5
Peptidyl-histidine phosphorylation	GO:0018106	3	0.035	9.7
Response to hydrogen peroxide	GO:0042542	4	0.039	5.2
Lipid transport	GO:0006869	9	0.049	2.2
Muscle development	GO:0007517	12	<0.1	1.8
Response to reactive oxygen species	GO:0000302	4	<0.1	3.8
Cellular lipid metabolic process	GO:0044255	32	<0.1	1.3
**GO Cellular Component**				
Mitochondrion	GO:0005739	56	0	1.5
Sarcoglycan complex	GO:0016012	3	0.027	11.1
**GO Molecular Function**				
Diacylglycerol kinase activity	GO:0004143	5	0	9.1
Pyruvate dehydrogenase (acetyl-transferring) kinase activity	GO:0004740	3	0.008	20.4
Oxidoreductase activity, acting on peroxide as acceptor	GO:0016684	5	<0.1	3.2
Electron donor activity	GO:0009053	2	<0.1	27.3
Phospholipid transporter activity	GO:0005548	4	<0.1	3.8

Gene ontology biological processes, cellular components and molecular functions that were significantly overrepresented among genes within selected regions and may be relevant to Thoroughbred selection. The complete list is given in Table S2. Selected regions were defined by deviation from expected heterozygosity (Ewen-Watterson test) in Thoroughbred and included the four high *F*_ST_ regions that had highly significant (*P*<0.01) reductions in Thoroughbred heterozygosity. The table shows the number of genes in each functional group, *P*-value for significance of over-representation and fold-enrichment.

We refined the complete list of genes in selected regions to a list of 87 candidate genes (85 had functional annotations on the *H. sapiens* GO database) that had the following (select) GO Biological Processes (containing more than five candidate genes): anatomical structure development; blood vessel development; carbohydrate metabolic process; carboxylic acid metabolic process; generation of precursor metabolites and energy; glucose metabolic process; G-protein signalling, coupled to IP3 second messenger (phospholipase C activating); insulin receptor signalling pathway; muscle development; positive regulation of catalytic activity; skeletal development and transmembrane receptor protein tyrosine kinase signalling pathway. Six KEGG pathways were among the list of candidate genes and included fatty acid metabolism, oxidative phosphorylation and regulation of actin cytoskeleton.

The *F*_ST_ statistic was used to identify genomic regions that distinguished Thoroughbred from non-Thoroughbred. Because *F*_ST_ may be influenced by unequal sample sizes, we limited our search for genes of interest to the nine high *F*_ST_ regions that also had significant deviations from expected heterozygosity in Thoroughbred. Gene ontology (GO) terms for Biological Processes returned 369 genes using the *H. sapiens* GO database. Genes with functions in 35 GO Biological Processes and 21 Molecular Functions (Table S3) were found significantly (*P*<0.05) more frequently in this gene list than expected for all searched (functionally annotated) genes (*n* = 15,360). GO terms that may have functional relevance for selection in Thoroughbred are listed in Table 3.

**Table 3:** Overrepresented gene ontologies (*F*_ST_).

GO Biological Process	GO Identifier	Number of genes	*P*	Fold enrichment
Spermatogenesis	GO:0007283	13	0.007	2.5
Cellular calcium ion homeostasis	GO:0006874	9	0.008	3.1
Porphyrin biosynthetic process	GO:0006779	4	0.009	8.8
Sexual reproduction	GO:0019953	16	0.001	2.0
Skeletal development	GO:0001501	12	0.022	2.1
Anatomical structure formation	GO:0048646	10	0.029	2.3
Hydrogen peroxide catabolic process	GO:0042744	3	0.032	10.4
Regulation of osteoblast differentiation	GO:0045667	3	0.037	9.6
Hydrogen peroxide metabolic process	GO:0042743	3	0.043	8.9
Mating	GO:0007618	3	0.043	8.9
Regulation of catalytic activity	GO:0050790	19	0.047	1.6
Insulin receptor signaling pathway	GO:0008286	4	<0.01	4.6
Muscle development	GO:0007517	9	<0.01	2.1
Response to oxidative stress	GO:0006979	6	<0.01	2.6
Actin filament organization	GO:0007015	4	<0.01	3.9
Detection of temperature stimulus	GO:0016048	2	<0.01	20.8
**GO Cellular Component**				
Mitochondrion	GO:0005739	34	0.025	1.5
Cortical actin cytoskeleton	GO:0030864	3	<0.01	6.7
**GO Molecular Function**				
G-protein-coupled receptor binding	GO:0001664	7	0.008	4.0
Oxidoreductase activity, acting on peroxide as acceptor	GO:0016684	5	0.014	5.3
Pyruvate dehydrogenase (acetyl-transferring) kinase activity	GO:0004740	2	<0.01	22.3
**KEGG Pathway**				
Focal adhesion	hsa04510	10	<0.01	1.8

Gene ontology biological processes, cellular components, molecular functions and KEGG pathways that were significantly over-represented among genes within selected regions and may be relevant to Thoroughbred selection. The complete list is given in Table S3. Selected regions were defined by the nine high *F*_ST_ regions that had significant (*P*<0.05) reductions in Thoroughbred heterozygosity. The table shows the number of genes in each functional group, *P*-value for significance of over-representation and fold-enrichment.

As for the selected regions identified by the Ewens-Watterson test, the complete gene list was refined to a candidate gene list of 74 genes (72 had functional annotations on the *H. sapiens* GO database) that had the following (select) GO Biological Processes (containing more than five candidate genes): alcohol metabolic process; blood vessel development; lipid metabolic process; muscle development; skeletal development; steroid metabolic process; and transmembrane receptor protein tyrosine kinase signalling pathway. Twelve genes localised to the mitochondrion of which seven were specifically localised to the mitochondrial inner membrane. Three KEGG pathways were found among the list of candidate genes and included Type II diabetes mellitus.

As well as genes within related categories, the candidate gene lists contained genes that had no known functional relatives. Genes acting alone may have a singular strong selective effect and therefore each candidate was considered individually. A full list of candidate genes for each selected region is given in Tables 4 and 5.

**Table 4:** Candidate genes in selected regions (Dh/sd).

Locus	Chr	Location EqCab2.0 (Mb)	Region delimited (Mb)	*n*	*ko*	*Ho*	*He*	S.D.	Dh/sd	*P*	*F*_ST_	*P*	No. genes in region	Candidate genes
*COR008*	9	18.9	16.9–20.3	128	9	0.437	0.820	0.041	−9.436	0.000	0.258	0.061	16	*ADHFE1, MTFR1*
*TKY316*	25	25.7	15.7–28.1	166	6	0.254	0.715	0.068	−6.775	0.000	0.352	0.020	67	*ATP6V1G1, GSN, HSDL2, LTB4DH, MUSK, NDUFA8, PTGS1, SVEP1, TNC, UGCG*
*TKY222*	4	38.6	34.4–40.1	216	5	0.133	0.656	0.085	−6.117	0.000	0.450	0.005	32	*ACN9, CALCR, C1GALT1, CYP51A1, DLX5, DLX6, DYNC1I1, PDK4, PON1, PPP1R9A, SGCE, SLC25A13*
*COR101*	18	59.1	54.2–63.5	178	8	0.563	0.790	0.047	−4.872	0.001	0.118	NS	33	*AGPS, ITGA4, ITGAV*
*LEX057*	3	36.3	31.6–41.2	210	6	0.384	0.717	0.070	−4.752	0.002	0.078	NS	53	*ADH6, COX4I1, CPNE7, CYBA, EIF4E, EMCN, FOXF1, GALNS, MANBA, MVD, SPIRE2, TSPAN5*
*HMS047*	22	40	37.6–46.0	190	5	0.273	0.657	0.086	−4.444	0.004	0.135	NS	46	*B4GALT5, BMP7, CYP24A1, DOK5, PCK1, PTPN1*
*TKY315*	27	20.8	20.1–21.5	216	6	0.393	0.713	0.074	−4.331	0.007	0.148	NS	12	*CNOT7, FGF20, MTMR7*
*UCDEQ425*	28	43.1	42.1–42.7	202	8	0.591	0.789	0.047	−4.244	0.003	0.098	NS	6	*CERK, PPARA*
*AHT059*	21	5.7	3.4–10.8	194	5	0.290	0.652	0.091	−3.956	0.005	0.160	NS	35	*MEF2B, NDUFA13, PIK3R1*
*Lex026*	X	19.4	18.6–21.5	118	5	0.324	0.665	0.088	−3.890	0.006	0.020	NS	9	*PCYT1B, PDK3*
*NVHEQ040*	11	9.1	4.1–10.5	202	6	0.426	0.714	0.075	−3.848	0.007	0.170	NS	77	*ACOX1, ATP5H, CYGB, EXOC7, FDXR, GALK1, GALR2, GRB2, PGS1, SOCS3, SPHK1*
*TKY359*	8	64.2	57.5–64.3	142	6	0.441	0.716	0.075	−3.686	0.010	0.130	NS	5	*PIK3C3, SYT4*
*AHT023*	30	22.5	18.8–23.4	202	4	0.150	0.566	0.114	−3.652	0.003	0.112	NS	16	*B3GALT2, GLRX2, TROVE2*
*ASB011*	19	45.2	40.9–45.8	216	6	0.463	0.715	0.069	−3.646	0.007	0.039	NS	24	*ATP6V1A, BOC, PLCXD2, SLC35A5,*
*NVHEQ079*	17	20.7	10.2–23.8	202	4	0.184	0.572	0.108	−3.602	0.007	0.380	0.018	79	*ALG5, DGKH, DIAPH3, EBPL, FOXO1A, HTR2A, LCP1, SLC25A15, SLC25A30, SMAD8, SUCLA2*
*UM176*	17	23.8	20.7–41.4	212	7	0.567	0.759	0.054	−3.560	0.007	0.185	NS	46	*DGKH, DIAPH3, EBPL, LECT1, SLC25A30, SUCLA2*
*AHT006*	4	34.4	32.3–38.6	180	7	0.552	0.759	0.058	−3.541	0.007	0.253	0.066	21	*CALCR, COL1A2, CYP51A1, PPP1R9A, SGCE, SRI*
*LEX007*	3	8.7	7.9–9.8	122	4	0.745	0.579	0.108	1.54	0.007	0.027	NS	29	*COX7B2, SGCB*

Candidate genes located within regions that have been subject to positive selection in Thoroughbred ranked by Dh/sd score; the locus with the strongest evidence for selection is listed first. Loci were localised to chromosomes and their positions were determined by *in silico* ePCR (Table S1). Each selected region was defined by loci flanking the central locus. The number of Thoroughbred chromosomes genotyped (*n*), number of observed alleles (*ko*), observed heterozygosity (*Ho*), expected heterozygosity (*He*), standard deviation (SD), deviation from expected heterozygosity (Dh/sd) and the associated statistical significance values (*P*) are shown. *F*_ST_ values are also shown to indicate inter-population differentiation at each locus. Three loci were also found among the highest *F*_ST_ regions listed in Table 5 (*TKY316*, *TKY222* and *NVHEQ079*). The number of genes (with *Homo sapiens* functional annotation) located within each defined region is given. Loci *NVHEQ079* and *UM176* are located adjacent on ECA17 and their defined regions overlap, resulting in five common candidate genes listed for both regions.

**Table 5:** Candidate genes in selected regions (*F*_ST_).

Locus	Chr	Location EqCab2.0 (Mb)	Region delimited (Mb)	*F*_ST_	*P*	Heterozygosity				Dh/sd	*P*	No. genes in region	Candidate Genes
						TB	AH	CON	TU				
*TKY355*	21	46.7	44.5–48.3	0.613	0.000	0.032	0.677	0.426	0.583	−1.034	NS	4	*DNAH5*
*TKY015*	1	71.2	66.5–76.4	0.452	0.003	0.274	0.542	0.535	0.616	−1.163	NS	39	*ACTA1, ACTN2, AGT, GGPS1, TOMM20*
*TKY222*	4	38.6	34.4–40.1	0.450	0.005	0.133	0.667	0.635	0.799	−6.117	0.000	32	*ACN9, CALCR, C1GALT1, CYP51A1, DLX5, DLX6, DYNC1I1, PDK4, PON1, PPP1R9A, SGCE, SLC25A13*
*COR001*	22	0.2	0–11.0	0.423	0.008	0.000	0.063	0.520	0.121	-	-	49	*ACSS1*
*AHT024*	5	12.2	7.8–21.8	0.404	0.010	0.153	0.633	0.500	0.514	−2.151	0.042	73	*ACBD6, ANGPTL1, ARPC5, CACNA1E, GLT25D2, LAMC2, NMNAT2, NPL, PRDX6, SERPINC1, SOAT1*
*TKY033*	11	36.8	21.9–39.7	0.392	0.013	0.030	0.606	0.616	0.544	−2.916	0.002	208	*ACACA, ATP5G1, B4GALNT2, CACNA1G, CACNB1, COX11, DGKE, DYNLL2, IGF2BP1, IGFBP4, LPO, MMP28, MPO, NEUROD2, OSBPL7, PCTP, PDK2, PHOSPHO1, PLXDC1, PPP1R1B, RSAD1, SGCA, SLC35B1, SOCS7, STARD3, STXBP4, TBX4, TCAP, TOB1, XYLT2*
*UM265*	10	55.8	55–57.8	0.388	0.015	0.243	0.556	0.750	0.634	−1.437	NS	14	*BVES*
*NVHEQ079*	17	20.7	10.2–23.8	0.380	0.018	0.185	0.752	0.613	0.714	−3.602	0.007	79	*ALG5, DGKH, DIAPH3, EBPL, FOXO1A, HTR2A, LCP1, RB1, SLC25A15, SLC25A30, SMAD8, SUCLA2*
*TKY316*	25	25.7	15.7–28.1	0.352	0.020	0.254	0.644	0.528	0.736	−6.775	0.000	67	*ATP6V1G1, GSN, HSDL2, LTB4DH, MUSK, NDUFA8, PTGS1, SVEP1, TNC, UGCG*
*AHT025*	8	2.6	0–14.4	0.319	0.023	0.245	0.574	0.627	0.595	−1.360	NS	133	*ACACB, ACADS, COX6A1, MTMR3, MTP18, MYO18B, MYO1H, SMTN,*
*COR040*	27	17.2	14.6–20.1	0.316	0.025	0.390	0.684	0.679	0.801	−1.654	NS	11	*PPP1R3B, SGCZ*
*UMNe197*	6	16.0	14.7–17.3	0.315	0.028	0.078	0.533	0.554	0.792	−2.571	0.014	13	*CCL20, COL4A4, IRS1*
*HTG009*	4	71.1	59.5–98.5	0.312	0.031	0.184	0.478	0.722	0.744	−1.788	NS	-	region too large (39 Mb) (includes *LEP*)
*COR032*	17	41.4	23.8–59.9	0.310	0.033	0.083	0.495	0.203	0.705	−2.569	0.013	-	region too large (36.1 Mb)
*TKY303*	18	31.1	23.1–31.9	0.309	0.036	0.064	0.671	0.443	0.688	−2.534	0.010	10	*ACVR2A*
*VHL150*	27	29.6	27.3–35.3	0.309	0.038	0.244	0.540	0.631	0.720	−2.863	0.017	17	*AGA, AGPAT5, ANGPT2, VEGFC*
*NVHEQ029*	4	4.5	3.1–7.2	0.305	0.041	0.413	0.578	0.786	0.869	−0.272	NS	25	*PBEF1, PMPCB, PRKAR2B*

Candidate genes located within regions that have been subject to positive selection in Thoroughbred ranked by *F*_ST_; the locus with the strongest evidence for selection is listed first. Heterozygosity values for Thoroughbred (TB), Akhal-Teke (AH), Connemara (CON) and Tuva (TU) are shown. Loci with lowest heterozygosity in Thoroughbred were included. Loci were localised to chromosomes and their positions were determined by *in silico* ePCR (Table S1). Each selected region was defined by loci flanking the central locus. Dh/sd scores and associated *P*-values for Thoroughbred are given to indicate within population deviation from expected heterozygosity. Four loci (*TKY222, TKY033, NVHEQ079* and *TKY316*) had highly significant (*P*<0.01) Dh/sd scores while five (*AHT024, UMNe197, COR032, TKY303* and *VHL150*) met a less stringent criteria (*P*<0.05). One locus was monomorphic in Thoroughbred and seven loci did not have significant deviations (*NS*) from expected heterozygosity in the Ewens-Watterson test. The number of genes (with *Homo sapiens* functional annotation) located within each defined region is given. Two regions (defined by *HTG009* and *COR032*) were considered too large (39 Mb and 36 Mb) to reasonably search for candidate genes, however *LEP* (Leptin), associated with obesity in mouse [137], is located at 83.4 Mb on ECA4. *COR032* is located adjacent to *NVHEQ079* and *UM176* on ECA17 together defining a 48 Mb region that may contain a number of strongly selected genes.

### Biological relevance of candidate genes

The region with the strongest signal for selection based on population differentiation (*F*_ST_ = 0.613, *P*<0.01) and low Thoroughbred heterozygosity was relatively gene-poor containing annotations for only four genes: *ANKH* (ankylosis, progressive homolog [mouse]), *DNAH5* (dynein, axonemal, heavy chain 5), *FBXL7* (F-box and leucine-rich repeat protein 7) and *TRIO* (triple functional domain [PTPRF interacting]). Considering the markedly lower heterozygosity in Thoroughbred (*Ho* = 0.032) compared to non-Thoroughbreds (Akhal-Teke *Ho* = 0.677; Connemara *Ho* = 0.426; Tuva *Ho* = 0.583) we expected to find a gene subject to a particularly strong selective effect during Thoroughbred development. The observed low heterozygosity in Thoroughbred was not a function of the small number of detected alleles (*n* = 2) as Connemara also had just two alleles. Mutations in dynein genes (*DNA11*, *DNAH5*, and *DNAH11*) have been linked to a common cause of male infertility in humans, asthenozoospermia, which is inherited in a dominant sex-specific manner [33]. Consequently, *DNAH5* may represent a good candidate for selection because of its role in male fertility. Among the nine high *F*_ST_ regions that also departed from expected heterozygosity in the Ewens-Watterson test, we found significant enrichments for genes in a number of functional categories relevant to reproduction including gamete generation, mating, sexual reproduction and spermatogenesis and genes in these categories may also provide strong candidates for selection for fertility traits.

The investigation of overrepresented functional ontologies revealed a significant enrichment for genes with functions in the phosphoinositide 3-kinase (PI3K) and insulin-signalling pathways in positively selected genomic regions in Thoroughbred. Insulin stimulates glucose transport to maintain glucose homeostasis via a number of transcriptionally active signalling pathways [34]. Among these the PI3K pathway plays a key role in insulin-stimulated glucose transport in skeletal muscle [35], [36], [37] via its interaction with IRS1 (insulin receptor substrate 1) [38] and its regulation by insulin of *PIK3R1* (phosphoinositide-3-kinase, regulatory subunit 1 [alpha]) gene expression [35]. In type 2 diabetes (T2DM) regulation by insulin of genes via the PI3K pathway is disrupted [39]. Among selected regions in Thoroughbred we found *PIC3C3* (phosphoinositide 3-kinase, class 3), *IRS1* and *PIK3R1*, transcripts of which are dysregulated in skeletal muscle from T2DM patients following stimulation with insulin [38], [40]. The insulin-receptor signalling pathway genes *FOXO1A* (forkhead box O1A), *GRB2* (growth factor receptor-bound protein 2), *PTPN1* (protein tyrosine phosphatase, non-receptor type 1), *SOCS3* (suppressor of cytokine signalling 3), *SOCS7* (suppressor of cytokine signalling 7) and *STXBP4* (syntaxin-binding protein 4) were also found in positively selected regions. The suppressor of cytokine signalling (SOCS) proteins have been implicated in the inhibition of insulin signalling by a number of mechanisms including inhibition of IRS [41]. As well as *IRS1*, genes in the T2DM KEGG pathway in selected regions included *CACNA1G* (calcium channel, voltage-dependent, T type, alpha 1G subunit) and *CACNA1E* (calcium channel, voltage-dependent, R type, alpha 1E subunit) within which polymorphisms have been linked with T2DM [42].

At the tail-end of the distribution for deviation from expected heterozygosity in the Ewens-Watterson test we found an extreme outlier microsatellite locus (*COR008*, Figure 1). Two candidate genes, *ADHFE1* (alcohol dehydrogenase, iron containing, 1) and *MTFR1* (mitochondrial fission regulator 1), were identified among 16 genes in this 3.4 Mb region. *MTFR1* encodes an inner mitochondrial membrane protein, previously known as CHPPR (chondrocyte protein with a poly-proline region), that promotes mitochondrial fission [43]. The expression of *MTFR1* in mice is highest in testes where it functions to combat oxidative stress [44]. Mitochondrial function and the management of reactive oxygen species are important for favourable exercise responses and therefore *MTFR1* may impose strong selection pressure in Thoroughbred by protection of mitochondria-rich tissue against oxidative stress.

The *ADHFE1* gene encodes hydroxyacid-oxoacid transhydrogenase [45], the enzyme responsible for the oxidation of 4-hydroxybutyrate (gamma-hydoxybutyrate, GHB), an energy regulator that promotes the release of growth hormone [46] during periods of sleep [47]. GHB has been used illegally as a performance enhancing drug by human athletes [48], [49]. The expression pattern and function of *ADHFE1* in adiposity may be relevant to its selection for exercise performance. In obese adipose tissue and following treatment of 3T3-L1 adipocytes *in vitro* with a PI3K inhibitor, LY294002 [50], *ADHFE1* transcripts are dysregulated [51]. *ADHFE1* is preferentially expressed in highly metabolic tissues and transcript expression is highest in brown adipose tissue [51]. The fate determination of common brown fat and muscle precursor cells into brown adipose tissue is controlled by the *BMP7* (bone morphogenic protein 7) [52] and *PRDM16* (PRD-1-BF1-RIZ1 homologous domain containing 16) genes [53]. Although we did not find evidence for selection adjacent to the *PRDM16* gene region (the closest microsatellite loci were located 7.4 Mb and 23.8 Mb either side), we found *BMP7* among selected regions in Thoroughbred. This locus had no evidence for selection in non-Thoroughbreds. This region corresponds to human 20q13.2–13.3, a well described obesity quantitative trait locus in mouse [54]. A number of receptor (*ACVR1B, ACVR2,*), signalling (*SMAD8*) and downstream target genes (*COX7B2*) of BMP7 were also among the selected regions. The bone morphogenic proteins (BMPs) signal via binding to a heteromeric receptor complex comprised of three type I and three type II transmembrane serine/threonine kinases [55] including *ACVR2A* (activin A receptor, type IIA). The receptor complex activates SMAD (mothers against decapentaplegic *Drosophila* homologue) pathway proteins that along with the p38 MAP kinase pathway are responsible for most of the functionalities of BMPs [56]. In the single outlier region at the positive end of the distribution for expected heterozygosity was *COX7B2* (cytochrome c oxidase subunit VIIb2) an isoform of the BMP7 target gene *COX7A1* (cytochrome c oxidase subunit VIIa polypeptide 1 [muscle]) [52]. In human populations BMPs and receptor complex genes (*BMP3, BMP5, BMPR2* and *ACVR1*) also have strong selection signatures [57]. The retinoblastoma protein, encoded by the retinoblastoma gene (*RB1*) has also been reported to be responsible for the control of adipocyte differentiation into white or brown fat tissue [58] and *RB1* was also among the genes found in the selected regions.

We detected one region that deviated very significantly from neutral expectations for both the *F*_ST_ and Ewens-Watterson tests that contained genes of particular importance, including *PDK4*. The expression of *PDK4* is co-ordinated by the transcriptional co-activator PGC-1α via ERRα (estrogen-related receptor alpha) binding [59]. PGC-1α, encoded by the *PPARGC1A* (peroxisome proliferator-activated receptor gamma, coactivator 1 alpha) gene, is a key regulator of energy metabolism that regulates insulin sensitivity by controlling glucose transport via SLC2A4 (solute carrier family 2 (facilitated glucose transporter), member 4; previously GLUT4), drives the formation of oxidative muscle fibres and co-ordinates mitochondrial biogenesis via its interaction with nuclear encoded mitochondrial protein genes [60].

The regulation of glucose utilisation is tightly controlled by the uptake of glucose by glucose transporters, the rate of glycolytic flux and the conversion of pyruvate to acetyl-CoA in mitochondria via the catalytic function of the pyruvate dehydrogenase complex (PDC). The critical rate limiting step in the oxidation of glucose is the regulation of assembly of the PDC which is controlled by pyruvate dehydrogenase kinase (PDK). PDK blocks the formation of the PDC resulting in the beta-oxidation of fatty acids to acetyl-CoA as the substrate for oxidative phosphorylation. Three genes (*PDK2*, *PDK3* and *PDK4*) of the four genes that encode PDK isoforms are located in the positively selected regions in Thoroughbred. Also, the *PDK4* gene promoter contains a binding site for the *FOXO1A* transcription factor, a key regulator of insulin signalling in liver and adipose tissue. Single nucleotide polymorphisms in *FOXO1A* have been found to have a protective effect on T2DM development and related phenotypes in humans [61]. *FOXO1A* was also found among the positively selected genomic regions in Thoroughbred and its *PDK4* promoter binding site sequence is conserved in horse. The transcription factors FOXO1 and SMAD have also been shown to be responsible for myostatin gene regulation and therefore play key roles in the regulation of muscle growth [62].

Hypoxia is an important stimulus for exercise-induced functional changes in skeletal muscle enhancing oxygen delivery and utilisation [63] by the activation of downstream genes of the master regulator of oxygen homeostasis HIF-1α (hypoxia inducible factor-1 alpha) [64]. In humans, sequence variation in *HIF1A* has been associated with VO_2max_ [65]. Angiopoiesis is one of the many adaptations effected by HIF-1α signalling [66], [67]. Three genes with roles in angiogenesis, *ANGPT2* (angiopoietin 2), *ANGPTL1* (angiopoietin-like 1) and *VEGFC* (vascular endothelial growth factor C) were among genes in the selected regions. Also, *ANGPTL3* (angiopoietin-like 3) was in the region defined by *AHT107* that was just outside the cut-off *F*_ST_ = 0.3, but was significant (*F*_ST_ = 0.279, *P*<0.05). The gene encoding HIF-1α (*HIF1A*) is located 7.2 Mb from the nearest microsatellite locus (*UM012*) at the proximal end of ECA24. Although *UM012* was marginally outside the highest ranked regions (Dh/sd = −2.9, *P*<0.05) in the Ewens-Watterson test it did have a significant (*P*<0.05) reduction in heterozygosity and also had a higher *F*_ST_ (*F*_ST_ = 0.215, *P* = 0.10) than six of the nine *F*_ST_ regions that had very significant (*P*<0.01) Dh/sd scores. Therefore, the genomic region containing *HIF1A* can be considered to have a moderate signature of positive selection. Another adaptation mediated by HIF-1α is the induction of glycolytic flux although we found no evidence for a significant abundance of glycolytic genes among the positively selected regions and no strong candidates were identified in this functional category. On the other hand, HIF-1α and PCG-1α have both been reported to induce COX4 (cytochrome c oxidase subunit 4) protein in human skeletal muscle [68], [69], [70].

Cytochrome c oxidase (COX) is a multi-subunit enzyme (Complex IV) that catalyzes the electron transfer from reduced cytochrome c to oxygen in mitochondrial respiration. Mitochondrial function is dependent on the proper assembly of electron transport chain complexes. Nuclear encoded COX4 is responsible for the regulation and assembly of mitochondrially encoded subunits of Complex IV on the inner mitochondrial membrane [69]. *COX4I1* was found among the positively selected regions in Thoroughbred along with nine other genes with roles in the KEGG oxidative phosphorylation pathway. In human skeletal muscle, *COX4* mRNA levels have been shown to be associated with mitochondrial volume and, by extension, VO_2max_ [71].

The most important role of mitochondria is the generation of ATP accomplished by the oxidative phosphorylation of acetyl-CoA arising either from glycolysis or fatty acid oxidation. We found one locus (*COR001*) that appeared to be fixed in Thoroughbred while retaining diversity in non-Thoroughbred populations; three alleles were sampled in Connemara. Notably, heterozygosity was also markedly low in Akhal-Teke. The region defined by this locus contained 49 genes, but only one good candidate for selection, the gene encoding acyl-CoA sythetase (*ACSS1,* acyl-CoA synthetase short-chain family member 1). Acyl-CoA synthetase has been suggested as a key control factor in the oxidation of fatty acids [72]. We also found a significant enrichment for genes with specific functions in fatty-acid metabolism and lipid transport among positively selected regions.

Since muscle plays a key role in Thoroughbred performance and may be particularly important to distinguish Thoroughbred [73], [74], we searched all high *F*_ST_ regions for genes with GO Biological Processes relevant to muscle structure and function including: adult somatic muscle development, muscle cell differentiation, muscle contraction, muscle development, muscle fibre development, muscle system process, myoblast differentiation, negative regulation of muscle development, regulation of muscle contraction, regulation of muscle development, skeletal muscle development, skeletal muscle fibre development, smooth muscle, smooth muscle contraction, somatic muscle development, striated muscle development and structural constituent of muscle.

Among the 20 muscle-related genes identified were *ACTA1* (actin, alpha 1, skeletal muscle) and *ACTN2* (actinin, alpha 2) that are located 2.8 Mb and 3.6 Mb respectively from *TKY015* that defined the region with the second highest inter-population differentiation (*F*_ST_ = 0.452, *P*<0.01) and comparatively low diversity in Thoroughbred (*Ho* = 0.274). Alpha actin protein is found principally in muscle and is a major constituent and regulator of the contractile apparatus [75], [76]. Mutations in *ACTA1* have been found to disrupt sarcomere function in patients with congenital fibre type disproportion [77] and other muscle weakness pathologies [78]. In skeletal muscle α-actinin is responsible for cross linking actin filaments between adjacent sarcomeres and is known to interact with PI3K [79]. Polymorphisms in the gene encoding α-actinin 3 (*ACTN3*) are among the best characterised athletic-performance associated variants in human endurance athletes [80], [81], [82] and evidence for positive selection in the genomic region surrounding *ACTN3* has been reported in humans [83]. While *ACTN3* is expressed principally in fast muscle fibres *ACTN2* is more widely expressed in skeletal and cardiac muscle. *ACTN2* is structurally and functionally similar to *ACTN3* and it has been suggested that *ACTN2* compensates function in the absence *ACTN3* [84]. Interactions between actin (and tubulin) and the cytosolic chaperonin CCT [85], [86] have been described suggesting co-evolution to facilitate protein folding [87]. *PCYT1B* (phosphate cytidylyltransferase 1, choline, beta), the gene encoding CCT-beta, was 600 kb from *LEX026* that defined a 2.9 Mb region that had a significant reduction in expected heterozygosity in Thoroughbred (Dh/sd = −3.89, *P*<0.01).

Finally, among the muscle-related genes in the high *F*_ST_ regions we found three (*SGCA, SGCE* and *SGCZ*) that encode transmembrane protein members (α, β, γ, δ, ε, and ζ) of the sarcoglycan complex [88]. A fourth sarcoglycan gene (*SGCB*) was found in the single region (*LEX007*) with evidence for balancing selection in the Ewens-Watterson test. The sarcoglycan complex is found associated with the dystophin-glycoprotein complex located at the sarcolemma of cardiac and skeletal muscle cells linking the contractile apparatus of the muscle with the lamina [89] thus providing a mechano-signalling role. Mutations in any one of the sarcoglycan genes destabilises the entire sarcoglycan complex [88]. Loss of sarcoglycan function leads to progressive loss of skeletal myofibres or cardiomyocytes such as that seen in patients with autosomal recessive Duchenne-like muscular dystrophy [90]. Mutations in *SGCE* cause myoclonus-dystonia syndrome [91] and penetrance is influenced by paternal expression of the gene [92], [93], while defects in *SGCA* cause limb girdle muscular dystrophy [94]. The dystrophin-glycoprotein complex contributes to the integrity and stability of skeletal muscle by its association with laminin receptors and the integrin-associated complex in the costamere [89]. Focal adhesion complexes form part of the costamere [for review see: 95]. Among the positively selected regions determined by high *F*_ST_ we found a significant enrichment for genes in the KEGG focal adhesion pathway. These genes included *TNC* (tenascin C), *TNN* (tenascin N), *TNR* (tenascin R), *LAMC2* (laminin, gamma 2), *COL1A2* (collagen, type1, alpha 2), *COL4A4* (collagen, type 4, alpha 4) and *ITGA3* (integrin alpha 3). *TNC* (tenascin C) may be particularly important for muscle integrity because of its role in the protection against mechanically-induced damage [96].

## Discussion

Intense artificial selection for racetrack performance has resulted in an exercise-adapted phenotype leaving signatures in the Thoroughbred genome that we have revealed using population genetic methods. Genes for exercise-related physiology as well as sexual reproduction seem to have been subject to the strongest selection pressures. We have identified a number of candidate performance genes that may contain variants that could distinguish elite racehorses from members of the population with less genetic potential for success. Revealing such polymorphisms may aid in the early selection of young Thoroughbreds in the multi-billion dollar global Thoroughbred industry.

The presence of genes for α-actinin, α-actin and its chaperon, CCT, and four of the six sarcoglycan complex genes as well as an overrepresentation of focal adhesion complex genes in positively selected regions in Thoroughbred suggest that selection for muscle strength phenotypes has played a major role in shaping the Thoroughbred. A better understanding of the role that these genes play in the strength and integrity of muscle may contribute to improved knowledge of mechanical sensing and load transmission [97] and may impact treatment of musculoskeletal disorders in humans.

Understanding genes selected for exercise-related phenotypes has potential to impact human medicine in other areas. One of the most notable findings was that positively selected genomic regions in Thoroughbred horses are enriched for insulin-signalling and lipid metabolism genes. There is a growing concern about the rapidly increasing incidence of obesity and its pathological consequences in the development of type 2 diabetes (T2DM) among human populations in the developed world [98]. Insulin is responsible for glucose homeostasis and insulin-resistance is a key feature of T2DM [99]. In obese individuals, skeletal muscle oxidative capacity and responsiveness to insulin are impaired [100]. Therefore, identifying and understanding the molecular functions of genes responsible for the control of fat metabolism and insulin sensitivity holds great promise for the development of pharmaceutical interventions for obesity and diabetes related health problems. In humans, exercise training is a common intervention to combat obesity [101] because physical exercise stimulates skeletal muscle insulin activity and glucose transport and the utilisation of free fatty acids as an energy substrate while promoting oxidative capacity by increasing mitochondrial mass and enhancing the development of oxidative muscle fibres [102].

Equine metabolic syndrome (EMS) is a recently described clinical disease in which horses develop insulin insensitivity similar to that described for T2DM in humans [103], [104], [105]. In the same way, management of EMS in horses requires a combination of exercise and dietary modification towards a reduction in calories and a substitution of carbohydrates with fat [104], [105], [106], [107]. During exercise, the rate of ATP generated to power muscle contraction is determined by the metabolic fuel available (carbohydrate and fat) which is either stored in the muscle (glycogen, triglycerides) or taken from the circulation (fatty acids, glucose) [108]. Certainly, the intensity and duration of exercise as well as diet will dictate the relative contribution of the different substrates to fuel metabolism. In horses, energy for low-intensity exercise is predominantly obtained from fat whereas energy for high-intensity exercise has a greater reliance on muscle glycogen [109], [110]. Although equine diets are typically high in carbohydrates and low in lipids, it has been found that chronic adaptation to fat-fortified feeds confers benefits to athletic performances of horses that may be due to enhanced insulin sensitivity and fat utilisation [103]. It has further been proposed that fat-enhanced diets may also sustain or enhance other signalling functions of insulin receptors on glycolysis and lipid utilization [111]. This possibility was supported by studies that found that the lactate threshold as well as the peak lactate (indicative of glycolytic activity) increased in Arabian horses adapted to a fat-enhanced rather than a sugar-enhanced diet [112]. Fat-adapted horses have been found to have faster times on the track [113] as well as longer run times to fatigue and higher peak plasma lactate concentrations [114].

Brown adipose tissue is distinct from white adipose tissue in its ability to expend energy and generate heat rather than as a lipid storage unit [115]. Until recently it was thought that brown adipose tissue occurred only in mammalian infants (and in rodent adults) but it is now thought that the metabolically active mitochondria-rich tissue may be retained in adults [116] and derives from a common precursor cell for muscle [52], [53]. The incidence of brown adipose tissue in young horses and its persistence in adult horses, to our knowledge, have not been reported. Positive selection for genomic regions containing genes such as *ADHFE1* that preferentially functions in highly metabolic tissues including brown adipose tissue, as well as two of the key determinants of brown fat cell fate, *BMP7* and *RB1,* and their receptors and signalling molecules, raises the intriguing question of whether Thoroughbred skeletal muscle retains brown fat-like properties, thus providing a selective advantage. The retention of brown fat into adulthood has been suggested as a mechanism to combat obesity [52], [53] and experimental increases in brown adipose tissue in rodents has been associated with a lean healthy phenotype [117].

The oxidation of fatty acids is highly efficient in the generation of ATP and is controlled by the expression of *PDK4* in skeletal muscle during and after exercise [118]. Equine *PDK4* is located in the genomic region that had the highest inter-population differentiation as well as a highly significant deviation from neutrality in the Ewens-Watterson test and is therefore one of the strongest candidates for selection for exercise adaptation. The location of the *ADHFE1* and *ACSS1* genes in two of the strongest selected regions as well as a 2.2-fold overrepresentation of lipid transport genes and an abundance of genes with specific lipid metabolism function among positively selected genomic regions suggests that Thoroughbreds have been selected for aerobic energy production increasing flux through fatty acid oxidation and electron transport. High concentrations of circulating fatty acids have a disruptive effect on insulin signalling pathways causing insulin resistance and the manifestation of T2DM in humans [119]. It has been reported that Thoroughbreds have an enhanced delivery of fat and glucose into skeletal muscle [103] and accumulate less fat than other horse breeds when fed the same diet [73], [120], which together may contribute to the naturally lean athletic phenotype for which they are renowned. The presence of genes that suggest a preference for the oxidation of fatty acids for energy production as well as insulin-mediated molecular signalling genes in the key selected genomic regions in a population that has been strongly selected for exquisite adaptations to exercise strongly supports the role of exercise in the prevention of obesity and the protection against T2DM. While rodent models for obesity and diabetes are well established [121], [122], here we propose Thoroughbred as a novel *in vivo* large animal model that may contribute to further insights into the complex molecular interactions that serve to protect against obesity and related pathological phenotypes that are influenced by exercise.

## Materials and Methods

### Horse Populations

#### Thoroughbred

A Thoroughbred is a registered racehorse that can trace its ancestry to one of three foundation stallions (*The Darley Arabian*, *The Godolphin Arabian* and *The Byerley Turk*) and one of the approximately 30 foundation mares entered in The General Studbook [123]. One hundred and twelve Thoroughbred horse samples were chosen from a repository (*n* = 815) collected with informed owners' consent from breeding, training and sales establishments in Ireland, Britain and New Zealand during 1997–2006. In all cases pedigree information was used to control for genetic background by exclusion of samples sharing relatives within two generations. Also, overrepresentation of popular sires within the pedigrees was avoided where possible. This work has been approved by the University College Dublin Animal Research Ethics Committee.

#### Non-Thoroughbreds

Fifty-two non-Thoroughbred samples were selected from two likely founder populations for the Thoroughbred: Akhal-Teke (*n* = 18), Connemara Pony (*n* = 17); and an ancient horse population, Tuva (*n* = 17). Akhal-Teke horses have been kept by Turkomen peoples in the geographic regions in and surrounding Turkmenistan for centuries and they may have contributed to the Thoroughbred through imported Eastern stallions (For example, *The Byerley Turk*). The Connemara Pony is an indigenous Irish horse population and was chosen to represent ancestral local horses of Ireland and Britain as Galloway and Irish hobby horses no longer exist. Tuva horses are an ancient horse population originating in the Republic of Tuva, geographically located in the southern Siberian steppe, one of the accepted centres of horse domestication [1].

### DNA preparation

Genomic DNA was extracted from either fresh whole blood or hair samples. Blood samples were collected in 7 ml Vacutainer K_3_EDTA blood collection tubes (Becton Dickinson, Franklin Lakes, NJ). Hair samples with visible hair roots were collected in airtight zip-lock bags. DNA was extracted using a modified version of a standard phenol/chloroform method [124]. DNA concentrations for all samples were estimated using a NanoDrop ND-1000 UV-Vis Spectrophotometer (NanoDrop Technologies, Wilmington, DE).

### Microsatellite markers

A panel of 394 equine microsatellites was genotyped; these loci were selected from the most recent horse genetic maps [125], [126], [127] to include markers, as far as possible, equally distributed across the 31 autosomes (ECA1-31) and the X chromosome (ECAX) with no *a priori* knowledge of neighbouring genes. The physical chromosome positions of the markers were localized by performing electronic PCR (ePCR) [128] using *in silico* primer pairs (Table S1) on the *Equus caballus* Version 2.0. (EquCab2.0) genome sequence (www.broad.mit.edu/mammals/horse/). Considering non-continuity among chromosomes there were 363 gaps between loci. Almost 85% of the gaps were <1 Mb with an average locus density of one every 5.7 Mb. The median distance between loci was 4.1 Mb. In the case of dog breeds, Pollinger *et al.* [17] suggest when loci are highly polymorphic then even a moderately distributed (one locus every 0.8 cM) set of loci is powerful to detect selected regions while minimising Type 1 error.

### Microsatellite genotyping

All polymerase chain reactions (PCR) were performed with an annealing temperature of 57°C in a 13 µl reaction with 50 ng DNA, 10×PCR buffer (Bioline USA, Randolph, MA), 150 µM each dNTP (Roche, UK), 0.5 U one of four fluorescent labelled tags (Dye Set DS-31 which contain blue dye 6-FAM, green dye VIC, yellow dye NED and red dye ROX labelled at the 5′ end with either forward or reverse primer) (Applied Biosystems, Foster City, CA), 1 U Immolase heat-activated thermostable DNA polymerase (Bioline USA, Randolph, MA), 0.1 µM forward primer and 0.25 µM reverse primer (Invitrogen, Carlsbad, CA) and 2.0 mM MgCl_2_ (Invitrogen, Carlsbad, CA). All PCR reactions were performed using the MJ Research DNA Engine® Dyad® thermal-cycler (MJ Research, Bio-Rad Laboratories, Waltham, MA) and involved initial denaturation at 94°C for 3 min, followed by 36 cycles of 30 s each at 94°C, 57°C and 72°C and final extension 10 min at 72°C. Aliquots of 1 µl PCR products amplified with four different colour dye labelled primers and three different product sizes were pooled together to reduce cost and increase efficiency. A total of 12 multiplexed PCR products were separated in one well by capillary electrophoresis using the ABI Prism 3700 fragment analyser (Applied Biosystems, Foster City, CA). Raw genotype data were analyzed using GeneScan software (Applied Biosystems, Foster City, CA) and alleles were then scored manually with each allele being assigned an integer value.

### Population genetic analyses

Knowledge of genetic variation under neutral expectations allows the identification of selection by assessing patterns that depart from those observed under conditions of neutrality [26]. Whereas population demographic effects will influence the entire genome, the key to this approach is that only a fraction of the genome will be subject to selection, and this can be detected as deviations from allele frequency expectations assuming neutrality. A number of statistical approaches can be applied to identify genomic regions that differ significantly from the remainder of the genome. Selection of advantageous allelic variants in certain populations can rapidly influence the frequency of alleles in a population, leading to differential allele frequency spectra at loci involved in the trait under selection, or loci linked by proximity to the selected variant [129], [130]. Inter-population differentiation resulting from allele frequency differences has traditionally been detected using the *F*_ST_ statistic [131], [132]. The spread of a beneficial variant also acts to reduce variability at a locus and its flanking regions, and this can be evaluated by assessing deviations from expected heterozygosity.

Inter-population differentiation, global *F*_ST_ [131], allele frequency, average observed heterozygosity (*Ho*) and the number of alleles sampled per locus were calculated using the software program FSTAT version 2.9.3 [133]. Statistical significance for each observed *F*_ST_ (*P* value) was calculated as the proportion of *F*_ST_ values from the empirical distribution that was greater or equal to the observed *F*_ST_ for that locus. For analyses of X chromosome loci, only female samples were included (Thoroughbred *n* = 72; Akhal-Teke *n* = 12; Connemara *n* = 17; Tuva *n* = 8).

To evaluate significant departures from expected heterozygosity, an indicator of positive selection, we calculated the Ewens-Watterson test statistic (Dh/sd) according to the Ewens formula [25], [134] for each locus in each population using the program BOTTLENECK [135]. The expected gene diversity (equivalent to Hardy-Weinberg expected heterozygosity) is calculated as a function of the observed number of alleles at a locus and the sample size and the difference between observed and expected heterozygosities is given as Dh. To normalise differences among microsatellite loci the Dh value is divided by the standard deviation (sd) of the gene diversity to give a Dh/sd value. Statistical significance (*P*) was assigned to Dh/sd values by completing 1,000 replicate simulations per locus and assuming a stepwise mutation model.

### Gene mining and functional annotation

Genomic regions, delimited by loci flanking each selected locus (Tables 4 and 5), were interrogated for genes annotated to the EquCab2.0 genome using the BioMart function in the Ensembl Genome Browser (www.ensembl.org).

Functional annotations (GO Biological Process, GO Cellular Component, GO Molecular Function and KEGG Pathway) were assigned for genes using the functional annotation tool, DAVID 2.0 [136]. Genes were clustered according to functional annotation using the Functional Annotation Chart command in DAVID 2.0.

## Supporting Information

Table S1 Equine microsatellite panel. Locus name and chromosome location for 394 equine microsatellite loci. The order on each chromosome is indicated and primer sequences given.
(0.08 MB XLS)

Table S2 Full list of gene ontologies overrepresented in selected regions (1). The on-line functional annotation bioinformatics tool DAVID (david.abcc.ncifcrf.gov) was used to search for gene ontology (GO) terms that were overrepresented among genes in selected regions. Selected regions were defined by deviation from expected heterozygosity (Ewen-Watterson test) in Thoroughbred and included the four high FST regions that had highly significant (P<0.01) reductions in Thoroughbred heterozygosity. The table shows the GO category (BP: Biological process; MF: Molecular function; CC: Cellular compartment; and KEGG pathway), GO functional term (Term), number of genes in each GO category (Count), proportion of the total number of genes in list in that GO category (%), significance of over-representation (P-value), the names of genes within each GO category (Genes), the total number of genes in the selected regions list (List total), the number of genes with GO term in the database (Pop hits), the total number of genes in the database (Pop total) and the fold enrichment (Fold change).
(0.13 MB XLS)

Table S3 Full list of gene ontologies overrepresented in selected regions (2). The on-line functional annotation bioinformatics tool DAVID (david.abcc.ncifcrf.gov) was used to search for gene ontology (GO) terms that were overrepresented among genes in selected regions. Selected regions were defined by the nine high FST regions that had significant (P<0.05) reductions in Thoroughbred heterozygosity. The table shows the GO category (BP: Biological process; MF: Molecular function; CC: Cellular compartment; and KEGG pathway), GO functional term (Term), number of genes in each GO category (Count), proportion of the total number of genes in list in that GO category (%), significance of over-representation (P-value), the names of genes within each GO category (Genes), the total number of genes in the selected regions list (List total), the number of genes with GO term in the database (Pop hits), the total number of genes in the database (Pop total) and the fold enrichment (Fold change).
(0.08 MB XLS)
